# Biodegradable Polymers-Based Smart Nanocrystals for Loxoprofen Delivery with Enhanced Solubility: Design, Fabrication and Physical Characterizations

**DOI:** 10.3390/polym14173464

**Published:** 2022-08-25

**Authors:** Barkat Ali Khan, Hina Khalid, Muhammad Khalid Khan, Khaled M. Hosny, Shahzeb Khan, Waleed Y. Rizg, Awaji Y. Safhi, Abdulrahman A. Halwani, Alshaimaa M. Almehmady, Farid Menaa

**Affiliations:** 1 Drug Delivery and Cosmetic Lab (DDCL), Gomal Centre of Pharmaceutical Sciences, Faculty of Pharmacy, Gomal University, Dera Ismail Khan 29050, Pakistan; 2 Department of Pharmaceutics, Faculty of Pharmacy, King Abdulaziz University, Jeddah 21589, Saudi Arabia; 3 Center of Excellence for Drug Research and Pharmaceutical Industries, King Abdulaziz University, Jeddah 21589, Saudi Arabia; 4 Department of Pharmacy, University of Malakand, Chakdara 18800, Pakistan; 5 Department of Pharmaceutics, College of Pharmacy, Jazan University, Jazan 45142, Saudi Arabia; 6 Departments of Pharmaceutics and Nanomedicine, California Innovations Corporation, San Diego, CA 92037, USA

**Keywords:** sustainability of natural resources, loxoprofen, smart nanocrystals, BCS II drugs, solubility enhancement, polymers

## Abstract

Nanocrystals are carrier-free, submicron-sized, colloidal drug delivery systems with particle sizes in the mean nanometer range. Nanocrystals have high bioavailability and fast absorption because of their high dissolution velocity and enhanced adhesiveness to cell membranes. Loxoprofen, a nonsteroidal anti-inflammatory drug belonging to the Biopharmaceutical Classification System (BCS) II drug class, was selected as the model drug. The aim of this study was to formulate nanocrystals of loxoprofen. A total of 12 formulations (F1 to F12) were prepared. An antisolvent technique was used to determine the effects of various stabilizers and processing conditions on the optimization of formulations. The various stabilizers used were hydroxypropyl methylcellulose (0.5%), polyvinylpyrrolidone (0.5%), and sodium lauryl sulfate (0.1%). The various characterizations conducted for this research included stability studies at 25 °C and 4 °C, scanning electron microscopy, transmission electron microscopy (TEM), X-ray powder diffraction (XRPD), differential scanning calorimetry (DSC), zeta potentials, polydispersity indexes, and dissolution studies. F10 was the optimized formulation that showed stability at room temperature, as well as at a refrigerated temperature, for 30 days. A high dissolution rate (100% within the first 10 min) was shown by comparative dissolution studies of nano-suspensions with the micro-suspension and raw loxoprofen. F10 formulation had a non-porous and crystalline morphology on evaluation by TEM and XRPD, respectively, and the average particle size was 300 ± 0.3 nm as confirmed by TEM. DSC recorded a reduction in the melting point (180 °C processed and 200 °C unprocessed melting points). The dissolution rate and solubility of the formulated loxoprofen nanocrystals were significantly enhanced. It can be concluded that selecting suitable stabilizers (i.e., polymers and surfactants) can produce stable nanocrystals, and this can potentially lead to a scaling up of the process for commercialization.

## 1. Introduction

The oral route of drug administration is the most acceptable one due to its safety and convenience [1] and patients prefer it, particularly over an extended time period [2]. However, conventional oral dosage formulations still face various pharmacokinetic and pharmacodynamic incompatibilities, which ultimately become hurdles in achieving therapeutic goals [3]. It is estimated that over 90% of the drugs approved since 1995 have various physicochemical problems, including poor aqueous solubility of drugs in their delivery to pharmacologically active sites [4]. Therefore, to achieve the effective oral delivery of drugs with low water solubility is often challenging because these drugs have an erratic bioavailability [5].

In the recent era of research, efforts to improve the solubility and dissolution of poorly water-soluble drugs are being employed; they include salt formulations, nanostructured lipid carriers, liposomes, nanoparticles, microparticles, solid dispersions, and nanocrystals (NCs). Drug NCs are pure solid nanoparticles covered by a stabilizing layer of a polymer [6].

In this study, NCs were investigated as drug carrier systems due to their high potential for drug loading. The NCs offered a significantly improved rate of dissolution, high saturation solubility, and enhanced bioavailability [7]. NCs resolved the solubility and low bioavailability issues. Additionally, they had significantly enhanced pharmacokinetic profiles, safety, efficacy, and potency [7,8,9,10]. NCs further gave us an advantage over adhesive forces, which become increased between reduced particles at their surfaces. The extended period and the enhanced adhesiveness provided a high absorption rate. Moreover, the NCs had a much higher dissolution time at the site of absorption, and this would be helpful for drug uptake. The nanoparticles adhered to the mucosal surface for an extended period, and this led to an enhanced rate of absorption of a drug [10,11,12].

Nonsteroidal anti-inflammatory drugs (NSAIDs) are among the most utilized drugs worldwide for the relief of pain, fever, and inflammation [10]. NSAIDs have their pharmacological effects by binding to cyclo-oxygenase (COX) to inhibit arachidonic acid, ultimately reliving pain [12].

Loxoprofen (LX), one of the NSAIDs, is a short-acting prodrug of phenyl mefenamic acid. It is a non-selective COX inhibitor with anti-inflammatory, analgesic, and antipyretic actions, which is classified as a Biopharmaceutical Classification System II (BCS II) drug because of its very poor aqueous solubility; it is very soluble in methanol, freely soluble in ethanol, and practically insoluble in diethyl ether, acetone, and chloroform [13,14]. The poor aqueous solubility of LX is the motivation for active research to produce a new dosage formulation that could efficiently provide improved solubility and, hence, bioavailability to achieve the therapeutic goals.

Considering all these points, the current study commenced with the fabrication of LX-loaded NCs and their subjection to all the necessary physiochemical tests available in the laboratory.

## 2. Materials and Methods

### 2.1. Materials

The active drug loxoprofen was purchased from Shanghai United Chemicals Co., Ltd. (Shanghai, China), and hydroxypropyl methylcellulose (HPMC) from Shin-Etsu Chemical Co., Ltd. (Tokyo, Japan), with type of substitution 2910, Grade 615, 15cp viscosity. The polyvinyl pyrrolidone (PVP K-30), batch no. 08297047GO, was purchased from BASF, Heidelberg, Germany, and potassium dihydrogen phosphate (KH_2_PO_4_) from Merck KGaA, Darmstadt, Germany. Sodium lauryl sulfate (SLS), batch no. 35880, sodium chloride (NaCl), and Pluronic 407 and sodium carboxymethyl cellulose (NaCMC) were all bought from Sigma Aldrich, St. Louis, MO, USA.

### 2.2. Methods

#### 2.2.1. Preparation of LX-Loaded NCs

The NC formulation was first optimized using varying concentrations of the active drug and other ingredients via the bottom-up technique as previously described by Khan et al. [9]. The optimized NC formulation was next subjected to particle size analysis and clarity and homogeneity evaluation. The bottom-up technique of antisolvent precipitation was used. The LX was steadily dissolved by adding a small quantity of powder in ethanol, 5 mg/mL, followed by 10 mg/mL, 15 mg/mL, 20 mg/mL, and 30 mg/mL to determine which maximum quantity of LX had the maximum solubility. The screening of the polymer was done with a range of stabilizers, including PVP K-30, HPMC, SLS, NaCMC and pluronic. The antisolvent solution consisted of 0.5% HPMC, 0.5% PVP, 0.3% pluronic, 0.1% SLS, and 0.1% NaCMC in distilled water. To formulate a stable nanocrystalline solution it was necessary to determine which series of precipitations in milliliters would work for a batch size of 2 mL. The amount of solvent solution (LX in ethanol) was 0.2 mL for a 2 mL batch-size solution, and the amount of antisolvent solution (stabilizer in water) was 1.8 mL. The LX solution (30 mg/mL) was injected into the antisolvent phase (stabilizer solution) and stirred at 1200 rpm for 60 min. After stirring, the suspension was ultrasonicated and the particle size was analyzed. Using centrifugation and washing three times with water, the dried nanoparticles were obtained. Finally, the drug-loaded NC was enclosed in aluminum foil and stored in the refrigerator at 4 °C. The compositions of the prepared NCs are shown in Table 1.

#### 2.2.2. Particle Size and Polydispersity Index

A zeta sizer (Malvern Panalytical Ltd., Malvern, UK) was used to study the particle size and distribution at 25 ± 1 °C. In addition, 10 µL of each formulation was shaken thoroughly with 1 mL of de-ionized water and vortexed for 2 min, and a zeta-sizer analysis was performed as previously conducted by Ndlovu et al. (2019) [15]. It is very important to emphasize the interactions and investigate the role of the polymer in the stabilization of drug NCs. It is therefore very important to screen the suitable polymer via systematic studies to find a nanosuspension with a homogeneous distribution of particles of the same size. Different stabilizers were employed to prepare antisolvent solutions for the nanoprecipitation of the LX. A stabilizer solution of 1% (w/v) was used as the antisolvent system. HPMC 0.5%, SLS 0.1%, NaCMC 0.1%, PVP 0.5%, and pluronics 0.3% were used. The average particle size and PDI were tabulated after the findings were recorded in triplicate.

#### 2.2.3. Zeta Potentiometry

For the surface charge measurement, zeta potentiometry was utilized. In practice, 700 µL of an experimental dispersion was poured into the zeta sizer’s cell, and the voltage was observed. In the aqueous phase of the prepared NC, the zeta potential was measured using the zeta sizer’s conductivity/electrophoretic mobility. Each measurement produced three mean values, which were recorded in triplicate. The higher zeta potential values were associated with higher repulsive forces among the particles, as previously reported by Du et al. [7].

#### 2.2.4. Particle Shape and Surface Morphology

SEM can be utilized to determine the particle size, particle shape, and morphology. The morphological characteristics of the pure loxoprofen and LX nanosuspension were evaluated by SEM and TEM, respectively [15]. The sample preparation involved using double-sided sticky tape to fix raw LX particles and calcium phosphate dibasic powder with absorbed NCs (recovered via filtration) on a metal stub. The Quanta 400 SEM (FEI Company, Altrincham, UK) was utilized to obtain photomicrographs of the LX samples at different magnifications. The SEM instrument was calibrated with the gold grid supplied with the instrument. Similarly, TEM (JEM-1200 EX, Jeol, Tokyo, Japan) was used to characterize the external morphology of LX nanocrystals. For this purpose, a drop of LX nanosuspension was placed on the copper grid surface and dried at ambient temperature and then magnesium uranyl acetate solution was applied as 2% aqueous solution for negative staining.

#### 2.2.5. Differential Scanning Calorimetry

DSC was used to determine the melting point of different samples and compare them to that of a reference material. At a predetermined rate, the reference material and sample were heated in a Q2000 DSC by TA Instruments, New Castle, DE, USA [16]. Indium was employed as the standard sample for calibration. LX, 0.5 to 5.5 mg, and NCs, 1 to 4.5 mg, were carefully placed inside an aluminum pan and crimped onto the pan with the central piece hole. The samples were heated at 10 °C/min with nitrogen gas, and 25 to 210 °C were fixed as the scanning range.

#### 2.2.6. X-ray Powder Diffraction

For the analysis of the samples, the diffractometer Bruker D8 (Germany) was used. Pure LX powder was first put into the plastic holder and then smoothed out with a glass rod. A sample disc of silicone was filled with a small amount of LX NCs recovered from the LX nanosuspension. The XRPD was calibrated using the corundum standard. The scanning was done over 5 to 50° 2 θ at a rate of 1° 2 θ/min using a copper Kᾳ radiation source with a wavelength of 1.5 Å and 1-mm slits. The samples were analyzed in triplicate.

#### 2.2.7. Stability Studies

To study the main aspects of the nanosuspensions, physical stability studies were conducted. The reported prime factor that affected particle growth in a nanosuspension was temperature [4]. Consequently, at two different temperatures (that is, a refrigerated temperature of 4 °C and a room temperature of 25 °C) the nanosuspensions were subjected to stability studies. The dynamic light scattering (DLS) technique was utilized to evaluate the particle size growth. Visual inspection was also carried out to observe changes in nanosuspensions. The particle size growth was determined over 30 days and plotted against different time intervals.

#### 2.2.8. Dissolution Studies

The rate of dissolution of the prepared nanosuspensions was compared with that of the LX microsuspensions and pure powder. The USP apparatus II (pedal method) was utilized as a standard method. The dissolution experiment in this project was performed with the DIS 6000 manufactured by Copley Scientific (UK) as reported by Jin-Seok (2019) and Khan et al. (2018) for nanoparticles with minor modifications [5,9]. The dissolution medium was a buffer solution of pH 7.5. The samples were put into 900 mL of the buffer in the dissolution vessels and kept at 37 ± 3 °C. After a specific interval of time, 5 mL of each sample was analyzed using a UV spectrophotometer at 2, 5, 10, 15, 30, 45, and 60 min. To maintain the volume of buffer in the dissolution vessels, 5 mL of fresh buffer was added after each sample was withdrawn. To ensure that the samples were completely dissolved, they were all filtered using a 0.2-m syringe filter.

#### 2.2.9. Statistical Analysis

Research data were analyzed using the average and standard deviation (SD). Various statistical tools (SPSS) such as the one-way analysis of variance (ANOVA) and student t-test were used. The *p*-value of less than 5% (*p* < 0.05) was considered statistically significant. All data were analyzed in triplicate.

## 3. Results and Discussion

### 3.1. Particle Size and Polydispersity Index

Different stabilizers impacted the particle size of LX, as shown in Table 2. Stabilizers were used individually and in combination. The Malvern Zetasizer (UK) was utilized to measure the particle size of each formulation. HPMC was useful in reducing the particle size up to a greater extent, as revealed by optimization experiments. However, the combinations were more effective than the individual components. The combination of HPMC 0.5%, PVP 0.5%, and SLS 0.1% in one experiment was found to be the most effective combination for decreasing the size of the particles; they were reduced to approximately 300 ± 3.0 nm with a PDI value of 0.3 ± 0.01. A PDI value of less than 0.5 indicates a homogeneous particle size distribution [4,16,17]. Furthermore, this is also predicted that HPMC, PVP, and SLS have strong absorbance on the surfaces of LX NCs. It has been reported that a mixture of cellulose polymers can efficiently encapsulate drug nanoparticles.

### 3.2. Morphology of LX-Loaded NCs

**Figure 1:**
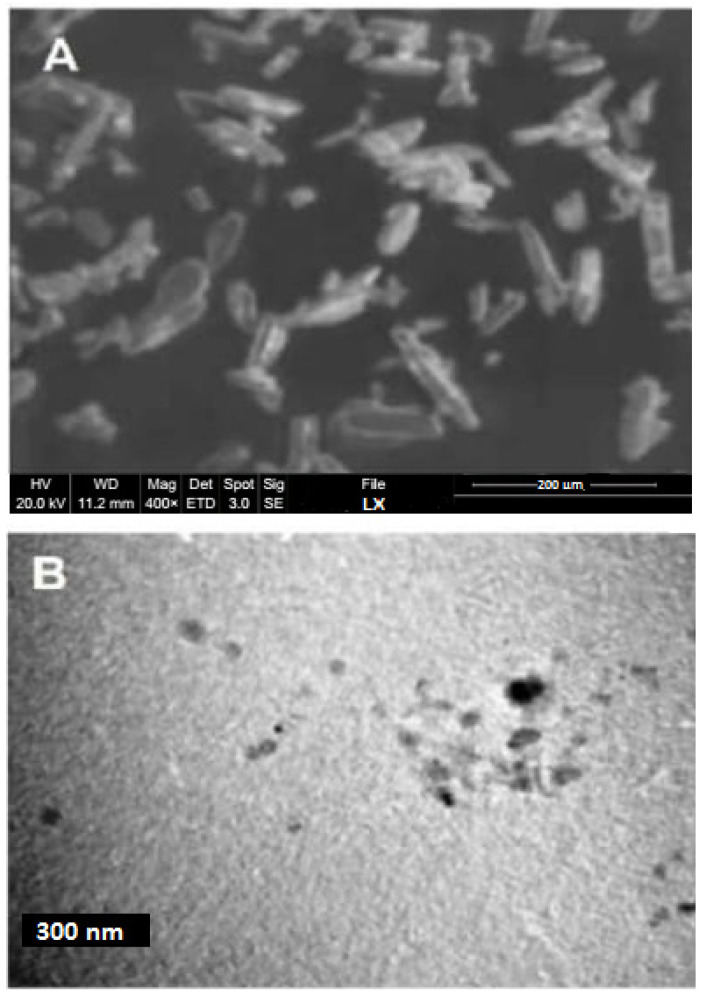
(**A**) Morphology of pure powder LX by SEM. (**B**) Morphology of LX NCS by TEM.

A scanning electron microscope (SEM) and transmission electron microscope (TEM) were utilized to evaluate the morphology of the pure and prepared LX samples, respectively. Pure LX particles were found to be crystalline in shape, as analyzed by the SEM at 500X magnification. The average particle size was 200 microns. Figure 1A shows the SEM image of the pure powder LX sample, and Figure 1B shows the TEM image of the prepared LX sample. The particles in Figure 1A have a variety of shapes, including plate, rectangular, and triangular, and their distribution is homogeneous. The prepared NC was analyzed by the TEM. The TEM images of the LX NCs, as shown in Figure 1B, were taken at 50 K, and the average particle size was 300 nm. The size was related to the DLC results. Because the zeta sizer was used, there was a small discrepancy in the size. The hydrodynamic layer around the particles was measured by DLS. TEM measures drug nanoparticles directly [18], and it confirmed the crystalline nature of the prepared samples and revealed that the sample preparation for the TEM and bottom-up process did not affect the particles’ morphological characteristics.

### 3.3. Differential Scanning Calorimetry

**Figure 2:**
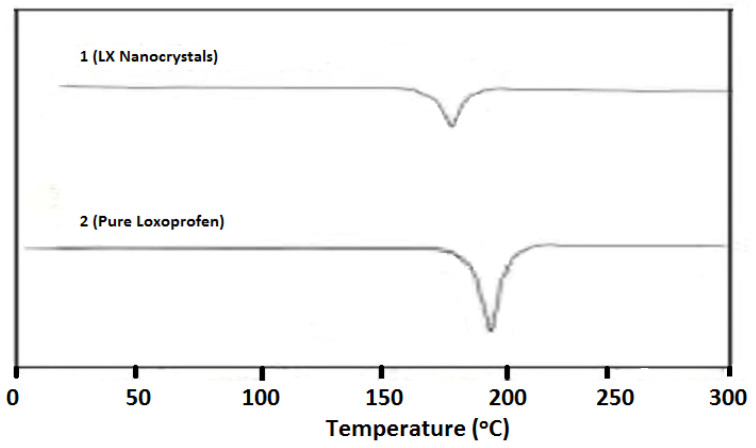
DSC studies of LX NCs (**1**) and pure powder (**2**).

To determine the internal packing density and morphology of the samples, especially powder samples, thermal characterization is important. The differential scanning calorimeter has been considered the most important tool for measuring the thermal profiles of microns and nanoparticles [4,17]. The information provided by this study showed how useful each sample might be in terms of its amorphous and crystalline nature [18,19]. The sharp peaks that appeared for the samples of the prepared LX and pure LX demonstrated the crystalline nature of both samples, as shown in Figure 2. Compared with the LX NCs, the peak for the pure LX particles was higher. The melting point of the pure LX was approximately 195 °C, and for the LX NCs, the melting point was reduced to 180 °C. In addition, the endothermic peak for the LX NCs was a little broader than the peak for the pure LX samples. The differences between endothermic peaks are due to particle size differences, as the particle size strongly affects the DSC profile. NCs had a relatively low enthalpy and melting point compared with the pure samples. The broadening of DSC peaks can be caused by impurities or traces of polymers remaining on the drug’s surface.

### 3.4. X-ray Powder Diffraction

**Figure 3:**
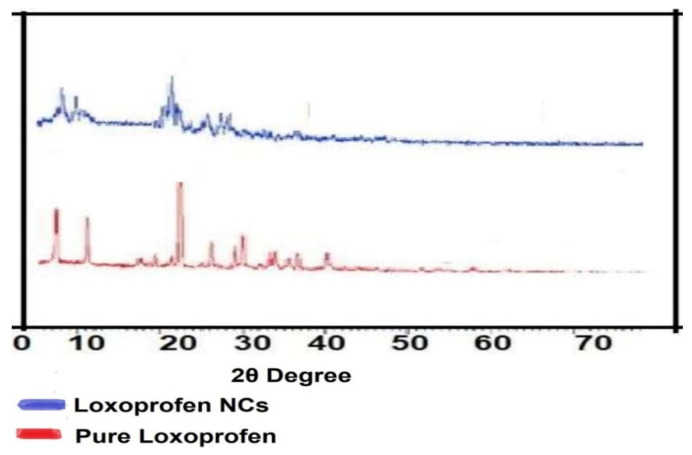
XRPD studies of LX NCs.

To evaluate the morphology and relative distribution function of the powder samples, XRPD analysis of the samples was performed. This helped in the determination of the different polymorphs of the drug samples. The relative distribution function studies disclose the packing arrangements of molecules in a compound crystal lattice, which could be helpful for the interpretation of the exact polymorphs of the compound [19,20]. Additionally, the diffractograms indicate the crystalline level of samples. Both samples were found to be crystalline in nature throughout this analysis, as indicated by their sharp peaks. However, the intensities of the peaks of LX NCs were relatively small and broad, and some of peaks had disappeared. This may be due to a small particle size, which can cause rays to be reflected at a narrow angle with a resulting peak of minor intensity [9,15]. The low packing density in LX NCs could also be a contributing factor to the absence of sharp peaks. Finally, the XRPD analysis found that the formulated LX NCs were crystalline in nature, although not as crystalline as the pure LX particles (Figure 3).

### 3.5. Stability Studies

**Figure 4:**
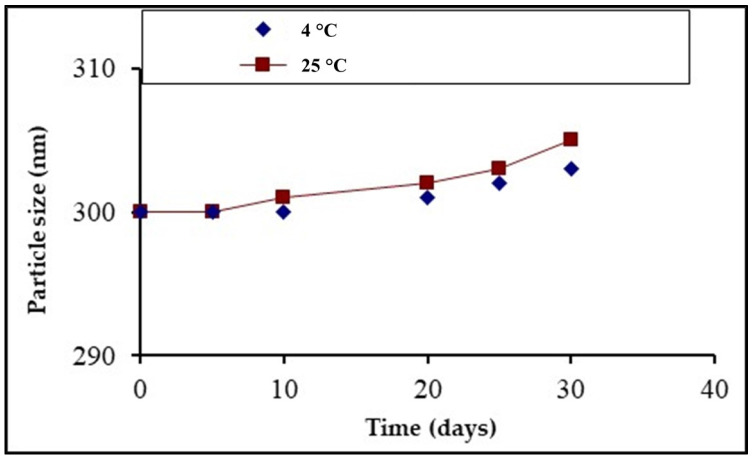
Comparative stability studies of LX NCs at different temperatures.

It is not a challenge to fabricate NCs but maintaining the stability of prepared NCs is a main issue. Nanosuspensions possess a great deal of free energy and are prone to the rapid aggregation and agglomeration of the suspended particles. For that reason, the formulated LX nanosuspension was subjected to stability studies for a period of 1 month at a room temperature of 25 °C and a refrigerated temperature of 4 °C, as shown in Figure 4. The appearance of the produced nanosuspension was monitored by visual inspection. At different time intervals, the particle sizes and PDI values were measured. PDI values are important because if the distribution of larger particles is not suitable, the particle growth can become accelerated [9]. The stability studies revealed that the nanosuspension particles stored at room temperature were less stable than the particles stored at refrigerated temperatures. Despite this, just a small quantity of growth was observed at room temperature. The nanosuspension produced by the bottom-up process had high energy owing to the common phenomenon of Ostwald ripening [21]. As a result, the selection of polymers and surfactants to be used is very important for stable nanosuspension production through the bottom-up process. The PDI values were less than 0.5 for both samples and this validated that both the samples were stable. The solubility recorded was very low at the refrigerated temperature, so the chances of dissolution of the particles were negligible and to migrate at the surface of large particles which leads towards Ostwald ripening. This part of the study revealed conclusively that both samples were stable.

### 3.6. Dissolution and Solubility Studies

**Figure 5:**
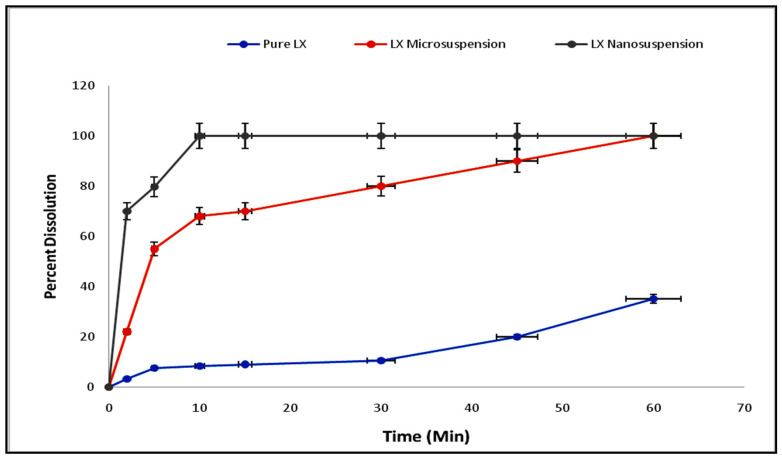
Comparative dissolution studies of pure LX, microsuspension of LX, and nanoemulsion of LX.

The dissolution study investigated the impact of the particle size on the dissolution profile of the fabricated NCs. It showed an increase in the rate of dissolution of the produced NCs relative to the pure powder of LX (Figure 5). In addition, it clearly revealed that within 5 min, approximately 80% of the LX nanosuspension had dissolved. A marked difference was observed between the rates of dissolution of the nanosuspension and pure LX particles. The higher dissolution rate of the LX nanosuspension was due to a difference in particle sizes. The rate of dissolution of the LX microsuspension was approximately 80% in 30 min, but a 100% rate of dissolution for the nanosuspension was attained within 10 min. Moreover, dissolution and solubility must be enhanced to attain a higher bioavailability [22,23]. The produced nanocrystals of LX maintained their size and surface properties, and as a result, they had higher rates of dissolution. NCs have a greater rate of dissolution and bioavailability owing to their small particle size and large surface area. According to the Noyes–Whitney equation, the dissolution rate is directly proportional to the surface area and inversely proportional to the thickness of the diffusion layer of particles [9]:

Dc/dt = ∆A/h(1)

where A indicates the surface area and h indicates the thickness of the diffusion layer of the particles. In the LX NCs, the surface area was increased but the thickness of the diffusion layer was decreased, and this led to an enhanced rate of diffusion [22].

## 4. Conclusions

This study demonstrated that the bottom-up process based on antisolvent precipitation is a cost-effective and efficient method of producing stable NCs. It provides a high level of micromixing with a transfer of the high mass, which leads to speedy formation of nanoparticles. Moreover, the solubility and dissolution studies revealed that the LX NCs displayed a marked increase in their rate of dissolution, which was due to a reduced particle size. Additionally, the appropriate selection of HPMC with PVP and SLS and the low concentration of the drug led to the maximum reduction in size of the particles.

## Figures and Tables

**Table 1:** Composition of drug-loaded NC formulations (2-mL batch size).

Sample	LX/Ethanol (mg/mL)	Distill Water (mL)	HPMC (w/v, mg/mL)	PVP (w/v)	NaCMC (w/v)	Pluronic (w/v)	SLS (w/v)
F1	6/0.2	1.8	0.009	----	----	----	----
F2	6/0.2	1.8	----	0.009	----	----	----
F3	6/0.2	1.8	0.009	0.009	----	----	----
F4	6/0.2	1.8	----	----	----	0.0054	----
F5	6/0.2	1.8	----	----	----	0.0054	0.002
F6	6/0.2	1.8	----	----	----	----	0.002
F7	6/0.2	1.8	----	----	0.002	----	----
F8	6/0.2	1.8	----	0.009	----	----	0.002
F9	6/0.2	1.8	0.009	0.009	0.002	----	----
**F10**	**6/0.2**	**1.8**	**0.009**	**0.009**	----	----	**0.002**
F11	6/0.2	1.8	----	0.009	0.002	----	----
F12	6/0.2	1.8	0.009	----	0.002	----	----

**Table 2:** Physicochemical evaluation of the prepared LX NCs.

Formulation Code	Size (nm)	PDI	Zeta Potential (mV)
F1	600 ± 4.0	0.8 ± 0.02	−16.0
F2	800 ± 5.0	0.9 ± 0.03	−14.5
F3	500 ± 4.5	0.7 ± 0.05	−15.2
F4	900 ± 6.0	0.6 ± 0.07	−12.0
F5	850 ± 7.0	0.7 ± 0.05	−13.5
F6	950 ± 7.5	0.8 ± 0.04	−11.2
F7	980 ± 6.0	0.5 ± 0.03	−11.0
F8	650 ± 5.5	0.9 ± 0.06	−16.0
F9	400 ± 4.0	0.4 ± 0.02	−18.5
**F10**	**300 ± 3.0**	**0.3 ± 0.01**	**−20.5**
F11	600 ± 4.0	0.5 ± 0.02	−15.0
F12	550 ± 6.0	0.6 ± 0.04	−14.8

## Data Availability

Not applicable.
